# H. A. Benham

**Published:** 1904-09

**Authors:** 


### Obituary.

### HARRY ARTHUR BENHAM, M.D.

The death of Dr. Benham, in his fiftieth year, on September 14th, 1904, has given rise to much regret throughout a large circle of medical, civic, masonic, and other friends and patients. He had been Medical Superintendent of the City and County Asylum at Stapleton for more than fourteen years, and previously was Assistant Medical Officer for a long period. His health had been failing for the last two years; his heart was known to be defective, so that a fatal syncope was not altogether surprising. He had great administrative ability, and exercised admirable control over a large and complex group of inmates of a class which are at all times difficult to guide. He took his degree of M.D. at Aberdeen in 1883, and has since devoted himself entirely to psychological work. He was Clinical Lecturer on Mental Diseases at the University College, Bristol, and a member of the Council of the Medico-Psychological Association of Great Britain and Ireland. He was an enthusiastic Freemason, had held many important offices in the Masonic Order, and was held in much esteem by a large number of Masonic friends, many of whom attended his funeral at Arno's Vale on September 17th, 1904. In his profession he took a leading position, and was frequently consulted on obscure forensic questions.

Dr. Lionel A. Weatherly writes as follows: "It was with feelings of the deepest regret and sadness that I heard the news of the death of my old friend, Harry Benham. I had been a fellow student with his deceased brother in 1870, and knew him then, but had no idea at that time that he intended to enter the medical profession. A few years after this he, however, decided that a doctor's life would suit him better than any other, and after studying at the Bristol Medical School, at the London Hospital, and at Aberdeen, he took the degrees of M.B., C.M. at that University in 1880. His first appointment was that of Assistant Medical Officer to the Dundee Royal Asylum, and soon afterwards he obtained the post of Assistant Medical Officer at the City of Bristol Asylum, with the late Dr. George Thompson as Superintendent. I well remember how quickly Dr. Thompson recognised the valuable helper he had in Dr. Benham, and when in 1890 ill-health caused his retirement, it was clear that no candidate for the office stood any chance against our friend, who at so early an age has passed away from our midst. The Committee can never have regretted the choice they then made, for in Dr. Benham they had a superintendent whose common sense, powers of organisation, clinical knowledge and humane feelings eminently fitted for the difficult work of presiding over such a large and important institution as the Asylum of the City and County of Bristol. Dr. Benham has seen this Asylum gradually grow and ever improve: the Committee would, I know, be eager to acknowledge how much they owe to the knowledge, energy, and love of his work of their Superintendent. All who had the happiness of being his friend will ever feel his death, and the medical profession of the West of England has indeed lost a valued member.

"The Medico-Psychological Association of Great Britain and Ireland was not slow to realise his worth, and he soon found a place on its Council. In 1899 he was appointed to the very important office of Registrar, and for some three years he carried out the duties to the fullest satisfaction of the Educational Committee and of the whole Association.

"Some two years ago his health compelled him to relinquish his work and take a long holiday. This he did, but when he came again amongst us it was evident that he was still in a very precarious condition. Bravely, however, he continued his work in the institution he so loved, to which he had so long given the best years of his life, and in which he peacefully fell asleep.

"He has gone from amongst us at a comparatively early age, but the memory of a true and kind heart, of a well-balanced and common-sense mind, will long remain ever present with all who could claim him as their friend."

A city alderman, who has long been on the Asylum Committee and has seen much of Dr. Benham's work, writes of him as follows: "By few will Dr. Harry Benham be more sincerely missed than the members of the Visiting Committee of that institution of which he was the able Medical Superintendent. When I first joined the Committee I was at once struck by Dr. Benham's courteous and genial manner, and during the whole time I knew him this never varied. But, speaking strictly from a committee-man's point of view, it was his administrative ability—which was of a very high order—that one most admired. It is difficult for an outsider to realise the innumerable matters, many of them of a difficult and complex nature, which have to be dealt with in an Asylum containing nine hundred patients and a necessarily large staff of nurses, attendants, and others; but day by day Dr. Benham did deal with these matters quietly, yet firmly, always with tact and good judgment, and so far as my experience goes invariably with the approval of the Visiting Committee. He was devoted to his work, and the welfare of the patients was his first consideration. A man of artistic tastes, he designed the decoration of the various wards in the Asylum, which decorations were carried out by the inmates with the most satisfactory results, and it was his constant care to see that everything about the wards was as bright and cheerful as good taste could make it. He grew a large number of plants and flowers, but only that his patients might enjoy them. The Corporation of Bristol has lost a faithful and valuable officer, and it is difficult to realise the committee-room at Fishponds without Dr. Harry Benham's familiar presence."

---

ARTICLES BY DR. H. A. BENHAM.

"Case in asylum practice where seven ribs were discovered to be fractured after death." *J. Ment. Sc.*, 1885-6, xxxi. 50–3.

BENHAM (H. A.) and J. GREIG SMITH: "Calculus on foreign body in bladder of an epileptic patient." *Bristol M.-Chir. J.*, 1886, iv. 38–42, 1 pl.

---

